# An exploration of moral hazard behaviors under the national health insurance scheme in Northern Ghana: a qualitative study

**DOI:** 10.1186/s12913-015-1133-4

**Published:** 2015-10-15

**Authors:** Cornelius Debpuur, Maxwell Ayindenaba Dalaba, Samuel Chatio, Martin Adjuik, Patricia Akweongo

**Affiliations:** Navrongo Health Research Centre, Navrongo, Ghana; INDEPTH Network, Accra, Ghana; University of Ghana, School of Public Health, Accra, Ghana

**Keywords:** Health insurance, Moral hazard, Kassena-Nankana District, Ghana

## Abstract

**Background:**

The government of Ghana introduced the National Health Insurance Scheme (NHIS) in 2003 through an Act of Parliament (Act 650) as a strategy to improve financial access to quality basic health care services. Although attendance at health facilities has increased since the introduction of the NHIS, there have been media reports of widespread abuse of the NHIS by scheme operators, service providers and insured persons. The aim of the study was to document behaviors and practices of service providers and clients of the NHIS in the Kassena-Nankana District (KND) of Ghana that constitute moral hazards (abuse of the scheme) and identify strategies to minimize such behaviors.

**Methods:**

Qualitative methods through 14 Focused Group Discussions (FGDs) and 5 individual in-depth interviews were conducted between December 2009 and January 2010. Thematic analysis was performed with the aid of QSR NVivo 8 software.

**Results:**

Analysis of FGDs and in-depth interviews showed that community members, health providers and NHIS officers are aware of various behaviors and practices that constitute abuse of the scheme. Behaviors such as frequent and ‘frivolous’ visits to health facilities, impersonation, feigning sickness to collect drugs for non-insured persons, over charging for services provided to clients, charging clients for services not provided and over prescription were identified. Suggestions on how to minimize abuse of the NHIS offered by respondents included: reduction of premiums and registration fees, premium payments by installment, improvement in the picture quality of the membership cards, critical examination and verification of membership cards at health facilities, some ceiling on the number of times one can seek health care within a specified time period, and general education to change behaviors that abuse the scheme.

**Conclusion:**

Attention should be focused on addressing the identified moral hazard behaviors and pursue cost containment strategies to ensure the smooth operation of the scheme and enhance its sustainability.

**Electronic supplementary material:**

The online version of this article (doi:10.1186/s12913-015-1133-4) contains supplementary material, which is available to authorized users.

## Background

Ghana has gone through a checkered health care financing history. Before independence in 1957, there were user fees at the point of health care access. After independence, health care became free and financed by general taxes and donor support [1]. However in the 1980s, there were economic crisis and sustainability of the free health care became questionable [2]. In the light of this, the government reintroduced user fees popularly known as “Cash and Carry” in 1992. The introduction of the user fees decreased the utilization of health care services at the public health facilities, and in 2002 alone about 80 % of Ghanaians needing health care could no longer afford it [1, 2]. The abolishment of the “Cash and Carry” became a topical issue and it became one of the key issues in the manifesto and campaigns of the main opposition party, the New Patriotic Party (NPP). Consequently, in fulfilment of the campaign promise, the NPP government upon winning the elections, established the National Health Insurance Scheme (NHIS) under Act 650 in 2003 [3].

Although pilot mutual health insurance schemes have been in existence in many parts of sub-Saharan Africa since the 1990s, Ghana remains one of the few African countries to establish a nationwide health insurance scheme. The scheme is being described in Ghana as the “passport to health care”. Essentially, the scheme is intended to replace out-of-pocket payments for health services through prepayment of insurance premiums. The health insurance scheme is seen as one of the pillars of the poverty reduction programme in Ghana. The NHIS is generally financed by a 2.5 % National Health Insurance Levy (NHIL) on selected goods and services, a 2.5 % Social Security and National Insurance Trust (SSNIT) deductions from the formal workers, premiums from the informal workers and non-SSNIT contributors, and government budget allocations. The informal sector workers would pay an annual minimum premium between Gh¢7.2 and Gh¢48 (approximately US$4.8 to US$32, using an interbank exchange rate of US$1 = GH¢1.5 as at November 2010) per person. Children below 18 years with at least one parent paying membership fees or covered by the exemption, pensioners who are formal sector contributors to SSNIT, the aged (70 years and above) in the informal sector and indigents are exempted from paying the premium. In addition, in 2008, the government of Ghana introduced a free maternal care policy that exempted all pregnant women from paying premium [4, 5].

Implementation of the national health insurance scheme required the establishment of mutual health insurance schemes at the district level. The NHIS has a minimum benefit package which every district mutual health insurance scheme (DMHIS) should cover. This package covers about 95 % of disease conditions prevalent in Ghana [5–8], including malaria, diarrhea, upper respiratory tract infections, skin diseases, hypertension, diabetes, and asthma. The package also includes services such as outpatient services, inpatient care and hospital accommodation, dental care, maternity care (including normal and caesarean deliveries), eye care, and emergency care. Some services considered very expensive such as cancer treatment (except breast and cervical cancer), organ transplant, dialysis , drugs not listed in the NHIS drugs list , assisted reproduction, private inpatient accommodation; non-vital services such as cosmetic surgery are not included in the benefit package [5–8]. HIV/AIDS symptomatic treatment for opportunistic infections has been added to the NHIS benefit package in 2012. There have been calls from various organizations to the government to let the NHIS cover the cost of antiretroviral drugs as many HIV/AIDS patients are faced with the risk of worsening conditions as they cannot afford the cost of the drugs. Nevertheless, HIV antiretroviral drugs are heavily subsidized by the National AIDS programme [6].

Studies have shown that health insurance schemes do improve access to health care and reduce out of pocket payments for services for insured individuals [5, 8–10]. Although less than half of the population of Ghana is currently enrolled in the NHIS [11], available records suggest that facility attendance has increased since the introduction of the scheme [12]. Despite the increase in access to health care due to the NHIS [13–17], there are reports of inequities in enrollment, with low enrollment among the poor [7, 18, 19].

Studies have examined various aspects of the NHIS in Ghana such as factors affecting enrollment [20–22], access to and quality of care [20], influence of NHIS on behavior of health care providers [16], and utilization of services [6]. Although these studies have broadened our understanding of the operation and impact of health insurance, various issues remain unclear. Among other things, the behaviors of insured persons and service providers which threaten the effective functioning of the scheme and the public’s awareness of these behaviors have not been adequately documented. In view of recent concerns by the National Health Insurance Authority (NHIA) that the soaring costs of health care for the insured is due in part to moral hazards from clients and service providers, it is important to examine the behaviors and practices of service providers and consumers (clients) that constitute moral hazards with a view to identifying ways to minimize such behaviors and enhance the sustainability of the Scheme.

Generally, client moral hazard behavior (demand side moral hazard) amounts to ‘taking advantage of’ insurance mainly because the individual does not have to make payment at the time of seeking health care services. On the other hand, provider moral hazard (supply side moral hazard or supplier induced demand) is over supply at the initiative of the health provider who takes advantage of the near absence of any financial considerations on the part of the patient if he/she agrees to buy the excess health care because cost will be transferred to the health insurance provider [23].

This study explored behaviors and practices of service providers and consumers of the national health insurance scheme in the Kassena-Nankana District (KND) that constitute moral hazards and identified strategies to minimize such behaviors.

## Methods

### Study site

This study was conducted in the Kassena-Nankana District of the Upper East region of Ghana. The district has a population of about 150,000 people in dispersed rural communities. There are two main ethnic groups – the Kassenas and Nankanas, with other ethnic groups forming about 5 % of the population. The predominant occupation is subsistence agriculture. As in many parts of the savanna zone of Ghana (where the district is located), poverty is endemic with poverty incidence of 88 % [24–26].

The Kassena-Nankana District is home to the Navrongo Health Research Centre, which operates the Navrongo Health and Demographic Surveillance System (NHDSS). As part of the NHDSS activities, households are routinely visited to monitor information about pregnancies, births, morbidity, mortality, migration, marriages, vaccination coverage and other vital socio-demographic variables [27].

Though the Kassenan-Nankana district has been divided into the Kassena-Nankana East and West districts since 2008, both districts were covered by one mutual health insurance scheme at the time of the study. For this reason we use the original name of the district – Kassena-Nankana District – to cover both districts.

The district has a hospital located in Navrongo that serves as a referral point for the Kassena-Nankana district, the Builsa district and neighboring towns in Burkina Faso. There are seven health centers, and two community clinics jointly run by the Catholic Diocesan Development Office and the District Health Administration that provide services to the communities. There is one private clinic and 27 functional Community-based Health Planning and Services (CHPS) compounds with resident Community Health Officers (CHOs) offering doorstep services [28].

The researchers are based in the Navrongo Health Research Centre (NHRC) located in the Kassena-Nankana District (KND). The district is a field site of the NHRC and our research intended to contribute towards strengthening NHIS in the KND. Given the limited resources for the research it was convenient to work within our own environment . This was an exploratory study and more manageable to do it in a familiar environment.

### Study design and data collection

This study was primarily qualitative, using in-depth interviews (IDIs) and focused group discussions (FGDs) to elicit information from community members, health care providers and managers of the Kassena-Nankana district mutual health insurance scheme. Interview guides (FGD and IDI) are presented as Additional file 1.

Eight individuals with Bachelor degrees (who are conversant in the local languages) were recruited and trained to collect data for this study. Actual data collection occurred between December 2009 and January 2010.

To explore community knowledge, perceptions and attitudes towards health insurance and behaviors that border on moral hazards, 14 focus group discussions (FGDs) were conducted with various categories of people in the district. Interviews were conducted among subgroups defined by gender, age and ethnicity. Participants in the FGDs were drawn from the catchment population of the main health facilities of the district.

The NHDSShas divided the Kassena-Nankana district into five zones - the East, West, North, South and Central zones. The East and South zones are predominantly Nankana speaking while the West and North are Kassem speaking (with the Central zone having a mixture of ethnic groups). The zones are further divided into clusters. We randomly selected three clusters in each zone as the location for the FGDs. Individuals who met the age (less than 30 years, 30–45 years, greater than 45 years), gender and ethnicity (Kassem and Nankana) criteria were then targeted for participation in the group discussion. Using the NHDSS database, a list of twenty eligible individuals in each category was generated and the first twelve eligible individuals who consented were invited to participate in each FGD.

In addition to FGDs, we conducted 5 individual in-depth interviews (IDI) with at least one medical assistant at the main health facilities to further explore issues of client behaviors from the providers’ point of view. Thus, medical assistants of the district hospital (the War Memorial Hospital), the Paga Health Centre, the Kassena-Nankana East Health Centre and the St. Jude Clinic were interviewed. These facilities represent the major public and private health facilities in the district.

Two in-depth interviews with KND health insurance scheme officers were also conducted to obtain further information on the insurance scheme and the behavior of clients and service providers.

### Data processing and analysis

The FGDs and IDIs were tape recorded, transcribed verbatim and typed. Guided by the objectives of the study and the themes of the discussion, a coding list was prepared to guide the data analysis. Thematic analysis was performed with the aid of QSR NVivo 8 software.

### Ethics and consent statement

The study protocol was reviewed and approved by the NHRC Institutional Review Board (NHRCIRB088) prior to study implementation. Informed consent to take part in the study was received from the participants.

## Results

### Moral hazard behaviors by insured members

A primary interest in this study is to identify behaviors exhibited by insurance scheme members that constitute moral hazards. Consequently FGD and IDI participants were asked about various behaviors that insured persons engage in that could compromise the efficient operation of the scheme. Various behaviors that amount to taking advantage of the NHIS (moral hazard behaviors) were disclosed by participants.

Frequent visit to health facilities by the insured was one such behavior identified. Respondents held the opinion that insured persons visit the health facilities more frequently compared to the non-insured. They also visit the health facility with the slightest ailment and even visit different facilities within the same period with the same ailment to collect more drugs.

*They feel cheated if they don’t go; once they are card bearing members any small cut on their toes, they are there (at the health facility to seek treatment). (FGD, Male).*

*Some of them when you give them the drugs within a day or two they are coming again without waiting for the drugs to work. Even we have detected that some immediately after they have collected the drugs will go to another facility with the same complaint and collect drugs again (Medical Assistant, Male).*

However, some FGD participants felt that insured persons were justified in going to health facility with the least ailment. According to them one runs the risk of incurring the anger of nurses when you wait for the situation to become serious before you go for medical care, as explained by this FGD participant:

*If you are insured and you happen to fall sick and you don’t go immediately for treatment and you wait till your situation worsens and you then go, the nurses will insult you and ask you why you are insured and you did not want to come early? (FGD, Female).*

Focus group participants also identified the practice of insured persons collecting drugs to keep at home. Insured people know that their membership will expire at the end of one year. So when the card is due to expire some who are not sick visit the health facilities to collect drugs to store at home. Some people feel cheated that they have paid to register and have not utilized the services. To avoid being cheated they go to the health facilities even if they are not sick so that they can use the services.

*Others too feel cheated by the scheme since they have not fallen sick since their registration, so they feel they should just go and collect the drugs (FGD, Female).*

*Every year in October cards will expire and so the hospitals are normally very full or the attendance is high because there are some people who will like to use their cards before they expire (NHIS official, Male).*

Another moral hazard behavior identified has to do with the insured persons using their cards to collect drugs for the uninsured. Respondents said that some people go to the health facility and describe the symptoms of their sick relations or friends who do not have the NHIS cards in order to get drugs to give to them.

In addition, the discussion participants revealed that sometimes the insured give out their cards to their sick relations or friends to go to a health facility to receive treatment*.* Some FGD participants however blamed the NHIS authorities for making it possible for such behaviors to occur. They stated that the pictures on the cards are sometimes blurred thus making it easy to impersonate.

*People who are insured sometimes give their cards to those who are not insured to go to the hospital for care. I however think that we need to blame the authorities for the success of this act because the pictures on some of the cards are so blurred that it is easy to deceive others that it is your card when it is not yours. (FGD, Male).*

Prescribers also mentioned the issue of the insured persons asking for particular drugs (most often expensive ones) to be prescribed for them. In response to the question whether health insurance members ask for particular drugs to be prescribed for them, one medical assistant had this to say:

*Yes, that one is big yes! Somebody will just come and ask you to write a certain drug for him/her. Sometimes when we tell them that we do not have that drug, they say we should just write it for them to go and collect at the pharmacy. I am always against that and because of that I do not have a friend here. (Medical Assistant, Female).*

Moral hazard behaviors are not limited to only the NHIS clients. There are various practices and behaviors by service providers that constitute moral hazards. We explored issues relating to reimbursement of service providers through interviews with NHIS officials to identify moral hazard behaviors. Some of the behaviors identified include over charging for drugs and services provided to clients, charging for services not provided, as well as inflating the number of clients provided with services.

*For now the payment to the health facilities is such that the more clients go to the facility the more we pay. Every client is by law supposed to visit the health facility three times within two weeks; that is first visit and the other two visits are for you to go for review. So within the two weeks, some clients can make one visit but once providers know that more visits attract more money they can take the folder and write the rest of the visits and charge accordingly. Some of these things may be funny but we get cases like that. (NHIS official, Male).*

Furthermore, the tendency of health providers to assign particular diagnosis to clients was mentioned. Specifically, it was pointed out that health providers tend to report simple malaria cases treated as severe malaria. The cost for treating severe malaria (GH¢89.00 or US$59) is higher than for simple malaria (GH¢28.00 or US$19); so some providers treat for simple malaria and charge the NHIS for severe malaria, or diagnose simple malaria as severe malaria.

*There was a time when every malaria that was reported was captured as severe malaria at the health facilities. But the issue is that we all know severe malaria and you cannot treat it at the OPD level and say it is severe malaria. The person comes and you give him/her treatment and he/she goes away and you say that is severe malaria, no. The person comes and you just detain the person for some few hours and you say that is severe malaria. So we had to talk to them and thank God at least it is better now (NHIS official, Male).*

Interviews with the scheme officials revealed that some NHIS officials condone with health providers to exploit the scheme. Over prescribing was also mentioned in the interviews as an issue that has the potential to affect the sustainability of the scheme.

*There were instances where one client could be given ten different types of drugs for one illness. We used to tell them (the prescribers) that it was not good and we at times tried to tell them that they are not supposed to do that. That is poly-pharmacy (giving so many drugs to a client). Even going by the Ghana Heath Service standard guidelines it says that you should not give more than five drugs for a particular illness. (NHIS official, Male).*

### Community perceptions on the effects of moral hazard behaviors

From the focused group discussions it was clear that community members are aware of the negative consequences of these moral hazard behaviors. Discussants indicated that such behaviors make health workers unwilling to treat insured people or give them good drugs when they go to the clinic because they think that they are just taking advantage of the NHIS.

Such behaviors have also discouraged really sick people with NHIS cards from going to the health facility, as they have to join unnecessarily long queues (due to large numbers of people attending the facility). Respondents indicated that such behaviors could lead to the collapse of the scheme as there will be less contribution and more utilization.

*Such behavior can cause the scheme huge debt and a time will come government will not be able to finance card bearing members and they can die out of that. (FGD, Male)*

*In fact, because many people go, they (health providers) don’t give them better drugs because they (health providers) also know it is because of the insurance people are just trooping in any how. (FGD, Female).*

### Suggestions to minimize moral hazard behaviors

FGD and IDI participants gave some suggestions that they felt if implemented would go a long way to discourage people from indulging in such morally hazardous practices. Some respondents were of the view that if the registration and renewal fees are reduced, people would find it easy to register themselves and their relations; this would reduce the need to give one’s card to an uninsured relative or to collect drugs for an uninsured relative.

*The government should reduce the registration fee. It is one of the causes why people don’t insure. If the amount is low everybody can be insured and the abuse of the scheme will be minimized drastically. (FGD, Female).*

Some respondents in the FGD and IDIs were of the view that provision should be made for people to pay their premium in installments.

*If you can get agents to always go round the community so that when the card is about expiring, we can be paying bit by bit so that by the time it is due for renewal we would have finished paying. (FGD, Female).*

FGD respondents were also of the opinion that the problem of impersonation could be minimized if health workers take their time to look at pictures on the cards well before attending to the patient. Also, those in charge of taking the photos should improve on their quality of work to make the pictures on the cards much clearer.

*I think the problem is always from the doctors because if they look critically, they will know the picture difference as well as the age. So government should have monitors who can from time to go round and monitor the behavior of such doctors and punish them.” (FGD, Male).*

The prescribers (medical assistants) interviewed suggested that the unnecessary visits by members could be reduced if the scheme could give a ceiling on the number of times a member could visit a health facility per month or per year.

*I would suggest that they should come up with a ceiling with regards to the number of times people could visit the health facility in a year. I think when they do this people will reduce the way they come to the health facility for treatment. For instance, if they say people under the health insurance scheme are supposed to visit the health facility two or three times in a year and beyond that you are supposed to pay, I think that will go a long way to address the issue of people coming for treatment very often. (Medical Assistant, Male).*

The NHIS officials emphasized education as a key tool that can change such morally hazardous behaviors from card members and health providers.

*“… on the part of the clients, I think the best way to address this situation is education. …. I think we cannot force them because some of the things that they do (like going to the hospital three times) because it is their right to go, you cannot force them. You can only educate them or convince them that the facility is for them and so they should not abuse it because when it collapses they are the ones who will suffer. On the part of the providers, I think education will help and again if there are prescribed punishment for providers who engage in such activities and offenders are punished, it will serve as deterrent to others” (NHIS official, Male).*

NHIS officials were of the view that when they have some training on the relevant drugs for treatment of some ailments it will help address the issue of over prescribing or wrong billing.

*Yes, I think that we the claims managers do not have the technical expertise and so sometimes you may not be able to tell whether the drugs given for a particular diagnosis are the right drugs or drugs that they should give for such diagnosis. The issue is that you know the symptoms for typhoid fever may be the same as that of simple malaria and since simple malaria is only GH¢28.00 (US$18.67) and that of typhoid fever is GH¢109.00 (US$72.67),when you identify a drug that is not supposed to treat typhoid fever and you draw their attention, you have a problem with them because they tell you what do you know and where have you done medicine. So these are some of the problems and so we really need to be given some training on some of these things (NHIS official, Male).*

## Discussion

The introduction of health insurance scheme is a major step in Ghana’s efforts to make health care accessible to the people of Ghana. By removing user fees payment at the time of seeking care, the financial barrier to health care access is removed thus making it much easier for people to seek care. Indeed, the increased attendance at health facilities since the introduction of the scheme strongly suggests that access to health care has been enhanced. However, the operation of the national health insurance scheme has faced various challenges which border on the behavior of insured persons, service providers and scheme officials. These abusive behaviors contribute to high health care expenditures that threaten the sustainability of the scheme.

The views expressed by community members in the focused group discussions highlight the significant impact of the national health insurance scheme in enhancing access to health care, especially for the poor. Nevertheless, some people are unable to pay the premiums and register with the scheme. The inability of some people to pay the premium or registration fees has been identified by other studies as a major barrier in enrolment into a health insurance scheme [5, 14, 15, 29–31]. Currently in the KND, all adult informal workers’ pay a premium of GH¢8.00 or US$5.3 in cash plus a registration fee or administrative charge of GH¢3/US$2 to join the scheme. Those who are exempted from paying the premium such as the SSNIT contributors and the aged (above 70 years) only pay the registration fee (GH¢3/US$2) to become a member. Though enrolments into the scheme over the past years have been increasing in the district, as at the beginning of 2012, only 50 % of the population in the district had valid membership cards [32]. Majority of those unregistered are the poor and vulnerable who are least able to pay for health care when they need it. It is therefore important to strengthen the exemption policy for the poor and vulnerable to enable them benefit from the scheme so as to achieve the equity goal of the NHIS and also accelerate universal health coverage.

Community members, health providers and NHIS officers are aware of various behaviors and practices that constitute abuse of the scheme (moral hazard). In the FGDs and in-depth interviews, behaviors such as frequent and ‘frivolous’ visits to health facilities, impersonation, collecting drugs for non-insured persons, over charging for services provided to clients, charging clients for services not provided and over prescription were identified. As expected, hardly any respondent admitted to personally engaging in these practices. Nevertheless, participants acknowledged that these practices exist and some even provided instances of people engaging in these practices. Other studies have reported moral hazard behaviors by clients such as the insured offering their cards to the uninsured to use to access care [16].

Various suggestions on how to minimize abuse of the NHIS were offered by community members, health providers and NHIS officials. Community members were of the opinion that a reduction of premiums and registration fees would make it possible for everyone to register and thus avoid the need to use someone’s card or collecting drugs for uninsured relations and friends. To further make it easy for people to pay their premiums some suggested that arrangements should be made for people to pay by installments. Regarding the problem of impersonation participants suggested an improvement in the picture quality of the membership cards as well as a critical examination of membership cards at health facilities to ensure that the photograph on the card is really that of the person attending the facility.

Currently, there is no limit to the number of times an insured person can visit an accredited health facility for care. Hence, to discourage people from unnecessarily attending health facilities some ceiling on the number of times one can seek health care within a specified time period was also suggested. Education was also identified as a key strategy in getting insured persons and health providers to change from behaviors that undermine the scheme. Generally, if people are well informed on the concept of social health insurance such as the Ghana NHIS and the in-built principle of cross-subsidization, moral hazard behaviors may change. For instance, with cross-subsidization, membership is based on ability to pay, and the rich will pay more while the poor pay less, thus the rich cross-subsidize the poor and vulnerable, the healthy cross-subsidize the sick [1].

The study also revealed some moral hazard behaviors by providers such as diagnosing simple malaria as complicated malaria, over charging for drugs and services provided to clients, charging for services not provided, inflating number of clients provided with services and over prescribing. NHIS officials suggested that when they have some training on the relevant drugs for treatment of some ailments it will help address the issue of over prescribing or wrong billing.

To address some of these moral hazards from the health care providers’ side and to reduce operational cost of NHIS, capitation payment mechanism is being contemplated to augment the current payment mechanism which is the Diagnostic Related Group (DRG). Initially, a fee for service type of provider payment mechanism was used to reimburse accredited health providers. However, this type of payment mechanism (fee for service) was reported to be low for providers to cover their cost of operation, especially the private providers and it also involved a lot of paperwork as they are required to provide detailed information on all services and charges for claims [16, 33]. In general, fee for service payment methods create the enabling environment for providers to provide unnecessary services to maximize profits [34]. In the light of these and other factors, in 2008, the system was replaced by the Ghana-Diagnostic Related Groupings (G-DRGs) [5, 16, 21]. The G-DRGs is a tariff system that groups diseases that are clinically similar, have comparable treatments or operations and use similar healthcare resources. With this system, the accredited providers are paid an already decided all-inclusive fixed payment for patient’s treatment according to their diagnostic group regardless of the costs [33]. The DRG for services also still holds some incentives for cost escalation though they are less than the fee for service. Because medicines at all levels continue to be under itemized fee for service, there is strong potential for moral hazard behaviors and consequently cost escalation [34].

Currently, the NHIS is piloting the capitation payment system. Capitation is a provider payment mechanism in which the health service providers such as physicians and nurses in the payment system are paid in advance a pre-determined fixed rate to provide a set of defined services for each enrolled person assigned to them for a period of time, whether or not the person seeks care [34]. This system aims at improving cost containment, controlling cost escalation by sharing risk between schemes, providers and subscribers, and improving efficiency in the use of resources [5, 35].

### Limitations of the study

This exploratory study was conducted in only the KND, out of the 216 districts in Ghana. Moreover, as a qualitative study it is not possible to estimate how prevalent the various behaviors and practices that undermine the scheme are in the KND. To the extent that the study district is not representative of all other districts in Ghana, the generalizability of the findings may be limited. However, given that the operations of the NHIS in the study district are not different from NHIS operations in other districts, the results obtained in this study provides useful insights on the situation in the other districts. Nevertheless, there is the need for further studies to cover more districts so as to identify moral hazard behaviors in other district which would inform the NHIS and how to address these issues to improve the operation of the scheme.

The NHIS is new in Ghana and it may well be that the behaviors identified in this study are associated with peoples’ lack of understanding of the concept of mutual health insurance. As people gain a better understanding and appreciation of the scheme some of these behaviors will disappear. Additionally, as the NHIA takes steps to improve on the efficiency of the NHIS it will become more difficult for clients and providers to engage in the various practices identified in this study.

## Conclusions

Ghana has taken a bold step in instituting the NHIS as a means to ensure universal health coverage. In barely a decade of its existence the scheme is believed to enhance access to health care. However, there are various challenges undermining the efficiency of the scheme that need to be addressed. Although the current premiums are considered to be low (in relation to the cost of medical care), community members observed that the premiums are high and so some people are unable to pay. In view of this, the policy within the scheme to enhance participation of the poor and indigents need to be applied rigorously. Also, appropriate sanctions and punishments for various forms of abuse of the scheme should be identified and rigorously applied. Punishing or sanctioning people who engage in behaviors that negatively affect the scheme will serve as a deterrent to others and help minimize the occurrence of such behaviors. On the other hand, mechanisms for rewarding individuals who regularly renew their membership for a specified time period without consuming health care services should be instituted.

## Additional file

Additional file 1:
**Interview guides.** (DOC 35 kb)

## Abbreviations

CHO: Community Health Officer
CHPS: Community-based Health Planning and Services
DMHIS: District Mutual Health Insurance Scheme
FGD: Focused Group Discussion
IDI: In-Depth Interviews
KND: Kassena-Nankana District
NHDS: Navrongo Health and Demographic Surveillance System
NHIA: National Health Insurance Authority
NHIL: National Health Insurance Levy
NHIS: National Health Insurance Scheme
NHRC: Navrongo Health Research Centre
NPP: New Patriotic Party
SSNIT: Social Security and National Insurance Trust
