# Supplementary material for: An exploration of moral hazard behaviors under the national health insurance scheme in Northern Ghana: a qualitative study
Source: BMC Health Serv Res. 2015 Oct 15;15:469. doi: 10.1186/s12913-015-1133-4 (PMC4606991; doi:10.1186/s12913-015-1133-4)
Supplement: Additional file 1: — Interview guides. (DOC 35 kb) [file 12913_2015_1133_MOESM1_ESM.doc]

# Interview guides

**Focus Group Discussion Guide**

**Navrongo Health Research Centre**

**An Exploration of Moral Hazard Behaviors under the National Health Insurance Scheme in the Kassena-Nankana District, Ghana**

**A. PREVENTIVE BEHAVIOURS**

- Is it possible to prevent sickness?
- What can individuals do to prevent sickness?
  - What do men do?
  - What do women do?
  - What do children do?
- What are the major difficulties or problems when someone gets sick?
  - Difficulties when an adult male gets sick
  - Difficulties when an adult female gets sick
  - Difficulties when a child gets sick

[Probe for issues relating to cost of treatment, affordability, etc.]

**B. KNOWLEDGE, MEMBERSHIP AND PERCEPTIONS OF MHIS**

- What is health insurance?
- Why the need for health insurance?
- Is there a health insurance scheme in this district? If YES, tell me about it.
  - Name of scheme
  - When did it start
  - Why was it started
  - Premiums and registration procedures
  - Membership categories
  - Benefits of membership
  - Types of services members can get
  - How often do you renew membership
  - Reasons for not renewing
  - Type of people who register and why
  - Type of people who have not registered and why
  - Compare membership fees to cost of treatment, which is greater

**C. MORAL HAZARD ISSUES**

- Does insurance membership encourage people to become sick? Why?
- Some people say that those who have insurance go to hospital ‘by heart’? Why does that happen?
- Some people who are insured go to hospital to collect drugs for their sick relatives or friends who are not insured. How often does this happen? Why? Is it right to do so?
- Some people who are insured sometimes give their cards to their friends or relatives who are not insured to go to the hospital. How often does this happen? Why? Is it right?
- You have registered with the scheme but as scheme member you have not fallen sick and the year is ending do you have to visit the health facility even though you are not sick?
  - If YES, Why?
  - If NO, why not?
- When you compare those who have insurance and those who do not, which of them go to hospital more often? Why is that so?
- At the health facilities do those who have health insurance receive the same services as those who have no insurance?
  - If NO, what are the differences? Which of them is treated better? Why are they treated better?
- Do you see any differences in the behavior of people who have health insurance compared to those who do not have insurance?
  - If YES, what are the differences?
- Have you noticed any changes in the way people go to hospital since the insurance was introduced?
  - If YES, what has changed?
- What are the things that people do not like about the health insurance scheme?
- What are the things that people like about the health insurance scheme?
- What can be done to make the health insurance scheme better?

**Discussion Guide for Health Workers Interview (IDI)**

**Exploration of Moral Hazard Behaviors under the National Health Insurance Scheme in the Kassena-Nankana District, Ghana**

**Introduction**

We are from Navrongo Health Research Centre (VAST). You are being invited to participate in a study on the National Health Insurance Scheme. Your participation will help us to learn about the operations of the scheme and the behavior of people who are insured. Your honest opinions about these issues will help us to formulate interventions and package educational messages tailored to make the scheme function more effectively. Your responses would be treated as confidential and will be used only for the purposes of this study.

Record the following information at the back of the cassette:

- Position of Interviewee e.g. Senior Nursing Officer, Medical Assistant, Doctor, etc
- Name of Health Facility
- Length of service at facility

**SECTION A: General Information on Health and National Health Insurance**

1. What are the main health problems that people bring to this facility?

Ask about problems that men bring

Ask about problems that women bring

Ask about problems that children bring

1. What kind of services do you offer in this facility?
2. How do people pay for the services they receive?

[Probe for cash payment, insurance, etc.]

1. I will like us to talk about the insurance:

When did it start?

What are the membership categories?

What are the health care services available for registered members?

Who decides on the service package for the insurance?

**SECTION: B Health seeking behaviors under insurance scheme**

1. What can you say about attendance at this facility before and after the introduction of the health insurance?
2. How has the health insurance influenced attendance at this facility?
3. In your opinion, are they differences in malaria cases before and after NIHS? Probe for severe and mild cases.
4. How do you charge for services offered at this facility?
5. Are the charges the same for insured and uninsured clients?

If No, why and what are the differences?

1. How do the non-insured pay for services?
2. What happens if a client cannot pay for services?
3. How do you recover the cost of services provided to insured clients?
4. What problems do you encounter in terms of payment for services?
5. What are some of the problems or challenges you face with the insurance system?

Probe for:

Work load

Expectations of clients

Cost of services

Payment for services

Etc.

1. What can be done to address these problems?

**SECTION C: Perceptions about health insurance**

1. How has the health insurance influenced the health behavior of people in this community?

Probe for:

Promptness in seeking care

Frequency of facility attendance

Preventive behavior

Adherence to therapy

1. What are some of the things that clients do that can affect the insurance scheme?

Probe for:

Using another person’s card to seek care

Using expired cards

Attending hospital to collect drugs for uninsured

Have you ever encountered any of these?

How often do these behaviors occur?

1. What can be done to minimize such behaviors?
2. Do registered members ask for particular drugs to be prescribed for them? Why.
3. Do you prescribe drugs based on NHIS membership? why.

- Probe for prescription of only paracetamol for the insured.

1. What are some of the things that health providers do that can affect the insurance scheme?

Probe for:

Prescribing expensive drugs

Selective billing of insured persons

Selective prescription

Given much attention to the uninsured

Etc.

How often do these behaviors occur?

What can be done to minimize such behaviors?

1. In your opinion, what do you think can be done to improve on the services provided in this facility?

**Additional Information**

1. Please, if there is anything you would like to add to our discussion, feel free to do so.

We will like to reassure you that whatever information you have volunteered in this discussion will be used for the purpose of the study and will remain confidential. We thank you very much for your time and participation in this study.
